# Hepatocellular carcinoma in pregnancy: A systematic review

**DOI:** 10.1111/aogs.14640

**Published:** 2023-08-02

**Authors:** Francesco Marasciulo, Irene Passerini, Anna Fichera, Federico Ferrari, Franco E. Odicino, Federico Prefumo

**Affiliations:** ^1^ Division of Obstetrics and Gynecology, ASST Spedali Civili, Department of Clinical and Experimental Sciences University of Brescia Brescia Italy; ^2^ Obstetrics and Gynecology Unit, IRCCS Istituto Giannina Gaslini Genoa Italy

**Keywords:** α‐fetoprotein, cirrhosis, hepatitis B virus infection, hepatocellular carcinoma, pregnancy

## Abstract

**Introduction:**

Hepatocellular carcinoma (HCC) is the most frequent primary malignant liver tumor and typically develops in the context of chronic liver disease, such as liver cirrhosis or chronic hepatitis B virus infection. Ultrasound evaluation, CT scan, and MRI are used to detect HCC. α‐fetoprotein (AFP) is a common marker used to detect HCC in the non‐pregnant population, which notoriously increases in pregnant women in relation to gestational age. Treatment is driven by the extent of the disease and the severity of underlying liver disease. Pregnancy may represent an obstacle to diagnosis and appropriate treatment of HCC. The aim of this descriptive systematic review was to describe the clinical features and maternal and neonatal outcomes of HCC in pregnancy.

**Material and methods:**

We performed a systematic review of the literature about HCC diagnosed in pregnancy and the postpartum period, with signs or symptoms arising in pregnancy. We included case reports and case series describing the clinical features of women diagnosed with HCC, fibrolamellar variant of HCC, and mixed HCC and cholangiocarcinoma during pregnancy or the postpartum period (with onset of symptoms during pregnancy), from inception to March 2023. The study protocol was registered with the PROSPERO database (Registration number: ID CRD42021275584).

**Results:**

We identified 180 records. The articles included in this systematic review were 47 case reports and 5 case series, for a total of 63 pregnancies. The two most frequent predisposing conditions were hepatitis B virus infection (30/63; 47%) and liver cirrhosis (14/63; 22%). Ultrasound evaluation was the most used technique to detect HCC. AFP was higher than normal in 28/46 patients tested (61%). Surgical treatment was the most used therapy, both during pregnancy and after delivery. Twenty‐six patients (26/63; 42%) died within 6 months of diagnosis. Survival >24 months was 9% (4/46) in symptomatic and 29% (5/17) in asymptomatic women. No patient with cirrhotic liver survived more than 12 months. Thirty‐eight newborns were alive at 28 days of age (38/63; 61%).

**Conclusions:**

Hepatocellular carcinoma in pregnancy is associated with a high risk of maternal and neonatal mortality. Diagnosis in asymptomatic high‐risk women or following abnormal maternal serum AFP screening is associated with better maternal outcomes.

## Graphical abstract

Hepatocellular carcinoma in pregnancy is a rare but severe condition, associated with a high risk of maternal and neonatal mortality. Incidental diagnosis in asymptomatic women with predisposing conditions or following abnormal maternal serum AFP screening may be associated with better maternal outcomes. Liver cirrhosis may be associated with a worse outcome and an overall survival less than 12 months.

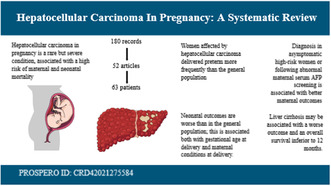

**Abbreviations**
AFP: α‐fetoprotein
HCC: hepatocellular carcinoma

**Key message**

Incidental diagnosis of hepatocellular carcinoma in asymptomatic women with predisposing conditions or following abnormal maternal serum AFP screening may be associated with better maternal outcomes. Liver cirrhosis may be associated with a worse outcome and an overall survival less than 12 months.

## 1 INTRODUCTION

Hepatocellular carcinoma (HCC) is the most frequent primary malignant liver tumor and typically develops in the context of chronic liver disease, such as liver cirrhosis or chronic hepatitis B virus (HBV) infection. In 2018, the estimated global incidence rate of liver cancer per 100 000 person‐years was 9.3 and the corresponding mortality rate was 8.5. The median age at diagnosis among women is 65–69 years.^1^ About 75% of primary liver cancers are HCC, and cholangiocarcinoma is the second most frequent neoplasm.^1^ HCC is more frequent in men than in women, with a ratio of about 3:1.^2^ The different sex distribution is thought to be due to differences in hepatitis carrier status, exposure to exogenous toxins, and/or potential effects of estrogens on interleukin‐6 (IL‐6).^3^ Inflammation is one of the major contributors to carcinogenesis, and IL‐6 is the multifunctional cytokine responsible for the hepatic response to systemic infections or inflammation.^4^, ^5^ Close ultrasound surveillance is recommended by major guidelines for patients at risk for HCC, liver cirrhosis, chronic active hepatitis B or C infection, and fibrosis. Biopsy in patients without liver cirrhosis and the use of CT or MRI in patients with cirrhosis allow early diagnosis even with nodules just greater than 1 cm.^6^ The best long‐term survival is observed with surgical treatment, resection and liver transplantation. However, several other non‐curative treatments are available for patients who are not candidates for surgery: percutaneous local ablation (thermal or by radiofrequency), transarterial chemoembolization and radioembolization and, rarely, local injection of substances, such as ethanol or acetic acid. Systemic treatments include target molecular therapies, immunotherapy and classical cytotoxic agents.^7^ The appropriate and tailored treatment is determined by the extent of the disease and the severity of underlying liver disease, which can limit tolerance to therapies. In this context, pregnancy can represent an additional challenge in the choice of proper treatment approach.

According to some authors, estrogens may mediate a protective effect through the inhibition of IL‐6 production in Kuppfer cells.^3^ It remains controversial, considering the small number of cases described in the literature, whether HCC observed during pregnancy differs from that arising in non‐pregnant women. In addition, pregnancy can represent an obstacle to diagnosis and appropriate treatment. Lau et al. in 1995 described a more aggressive behavior of HCC during pregnancy,^8^ suggesting that pregnancy is an adverse factor for the prognosis of HCC. However, a recent literature review has described an improvement in HCC‐related mortality and morbidity during pregnancy, due to improved diagnostic and therapeutic capacity.^9^ One of the serum markers most commonly used to detect HCC in the non‐pregnant population is α‐fetoprotein (AFP),^10^ which notoriously increases in pregnant women in relation to gestational age.^11^ This marker can be used to detect possible fetal abnormalities, and for this purpose is generally measured before 20 weeks of gestation.^11^

The aim of this descriptive systematic review was to describe the clinical features and maternal and neonatal outcomes of HCC in pregnancy.

## 2 MATERIAL AND METHODS

This was a systematic review of the literature on HCC diagnosed in pregnancy and the postpartum period, with signs or symptoms arising in pregnancy.

### 2.1 Data sources

A literature search was performed in the electronic databases PubMed, MEDLINE, Embase, and Cochrane Library from their inception until March 2023. For the purpose of this study, the research included combinations of the following terms: (pregnancy[Title]) AND (hepatocellular carcinoma[MeSH Terms]), (hepatocellular carcinoma[Title]) AND (pregnancy[Title]). The research aimed to identify all articles published in English, French, Spanish, or German until March 2023, that report cases of HCC arising during pregnancy or the puerperium.

### 2.2 Main outcomes measures

Clinical features, diagnostic and treatment approaches, and fetal and maternal outcomes.

### 2.3 Eligibility criteria

Given the rarity of HCC in pregnancy, we included case reports and case series describing the clinical features of women diagnosed with HCC, the fibrolamellar variant of HCC, and mixed HCC and cholangiocarcinoma during pregnancy or the postpartum period (with onset of symptoms during pregnancy). Studies with a presumptive diagnosis of HCC were included, even without certain histological data. To assess the methodological quality and risk of bias, we used the tool reported by Murad et al.^12^ based on the domains of selection, ascertainment, causality, and reporting.

### 2.4 Data collection and analysis

The titles and/or abstracts of the identified studies were screened independently by two authors (IP and FM). Subsequently, the same authors recovered the entire texts of screened papers and assessed the eligibility. The reference lists of all identified articles were systematically reviewed to identify other eligible publications. For all unavailable articles or incomplete data, we contacted the corresponding authors. Every disagreement on the eligibility of the data was resolved with consensus. The systematic review was performed following PRISMA guidelines. From each study, the following data were extracted: maternal ethnicity, clinical features, histological data, maternal serum AFP concentrations, coexistence of previous chronic liver disease (cirrhosis and chronic hepatitis virus infection), gestational age at diagnosis, diagnostic and treatment approaches, gestational age at childbirth, maternal and neonatal morbidity and death. Both IP and FM assessed the methodological quality of all the papers.

For statistics, we used descriptive analysis. Comparative analyses were not performed because of the small number of patients, spread over a large period of time.

The study protocol was registered with the PROSPERO database (Registration number: ID CRD42021275584).

## 3 RESULTS

### 3.1 General characteristics of the studies

**FIGURE 1:**
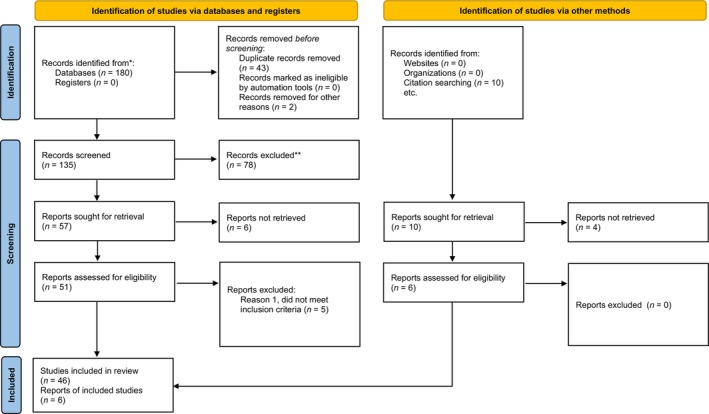
PRISMA 2020 flow chart and steps of studies selection. Citations of excluded studies are provided in Table S3.

We identified 180 records and screened 135 articles for evaluation. We excluded 78 records, and then analyzed the remaining 57 articles, from which we excluded 5 other records that did not meet the inclusion criteria, leaving 52 articles that met the inclusion criteria. The articles included in this systematic review were 47 case reports and 5 case series, for a total of 63 pregnancies. The study selection process is shown in Figure 1. The characteristics of each patient are summarized in Table S1. The assessment of the quality and risk of bias of each article is summarized in Table S2. The citations of the excluded studies are listed in Table S3.

### 3.2 Synthesis of the results

From the records, we extracted data of 63 pregnant women. Maternal characteristics are summarized in Table 1. The two most frequent comorbidities were HBV infection (30/63; 47%) and liver cirrhosis (14/63; 22%). We included 25 women from East Asia (40% of the sample), of whom 23 had a previous diagnosis of HBV infection (23/25; 92%).

**TABLE 1:** Epidemiology and characteristics of pregnant women affected by hepatocellular carcinoma.

Maternal characteristics	All (*n* = 63)	Symptomatic (*n* = 46)	Asymptomatic (*n* = 17)
Median age at diagnosis (17–45 y)	30	30	31
Country of origin			
Caucasian	16 (25%)	13 (28%)	3 (18%)
African	11 (17%)	10 (22%)	1 (6%)
South Asia	6 (10%)	4 (9%)	2 (12%)
East Asia	25 (40%)	14 (31%)	11 (64%)
Latin America	2 (3%)	2 (4%)	
Middle East	1 (2%)	1 (2%)	
Not known	2 (3%)	2 (4%)	
Previous diseases			
HBV	30 (47%)	18 (39%)	12 (71%)
Cirrhosis	14 (22%)	11 (24%)	3 (18%)
Imaging			
Ultrasound	48	32	16
CT scan	8	6	2
MRI	20	12	7
Diagnostic surgery	2	2	0
RX	4	3	1

Abbreviations: CT, computed tomography; HBV, hepatitis B virus; MRI, magnetic resonance imaging; RX, X‐ray.

Three women (3/63; 5%) had a previous diagnosis of HCC, and one (1/63; 2%) had a diagnosis of recurrent HCC during pregnancy (all of them unplanned). One of them terminated the pregnancy; one had a vaginal birth at 33 weeks of pregnancy; and the last one died during hospitalization before any treatment could be performed.

Ultrasound evaluation of the liver was the most used diagnostic technique (48/63; 76%), followed by MRI (20/63; 32%) and CT scan (7/63; 11%).

**FIGURE 2:**
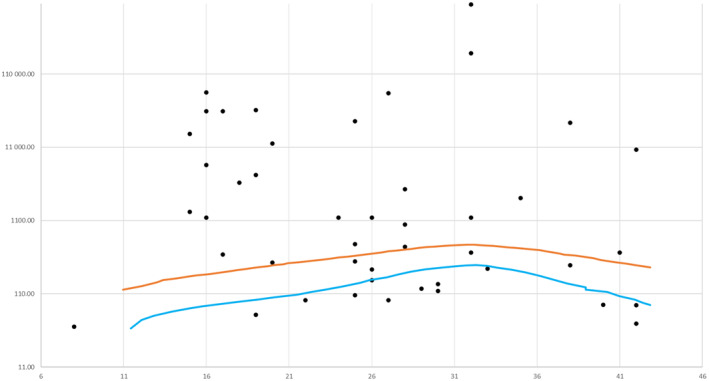
Maternal α‐fetoprotein (AFP) serum concentrations in pregnant women with hepatocellular carcinoma. The blue and orange lines represent lower and higher normal values of AFP levels in pregnancy, derived from Hay et al. 1976.^11^

Maternal serum AFP concentrations, known for 46 women (46/63; 73%), are shown in Figure 2. In 28 women, AFP concentrations were higher than normal (28/46; 61%); in 18 women the values were in the normal range or below limits (18/46; 39%).

The signs and symptoms of HCC are several, and include abdominal pain, ascites, gastrointestinal symptoms, jaundice, hypoglycemic coma, hemoperitoneum, rectal bleeding, and hemorrhagic shock.

The gestational age at HCC diagnosis was different in women with symptoms (17/63) and in asymptomatic women (46/63), with higher prevalence of women without symptoms and with high AFP serum levels receiving diagnosis between 10 and 19 weeks (9/17; 53%), and a higher prevalence of women with symptoms (especially abdominal pain) receiving diagnosis in the second and third trimesters (32/46; 69%). In the symptomatic group, 2 women received diagnosis before 9 weeks of gestation (2/46; 4%), 5 women received diagnosis between 10 and 19 weeks (5/46; 10%), 16 women received diagnosis between 20 and 29 weeks (16/46; 35%), and 16 women received diagnosis after 30 weeks of gestation (16/46; 35%). One woman without symptoms received diagnosis before 9 weeks (1/17; 6%), nine women received diagnosis between 10 and 19 weeks(9/17; 53%), five women received diagnosis between 20 and 29 weeks (5/17; 29%), and two women received diagnosis after 30 weeks of gestation (2/17; 11%). In the symptomatic group, 16 women had a vaginal birth (16/46; 35%), 21 women underwent a cesarean section (21/46; 46%), three women had a miscarriage (3/46; 6%), three women died before delivery (3/46; 6%), and one decided to terminate the pregnancy (1/46; 2%). Three women without symptoms had a vaginal delivery (3/17; 18%), seven had a cesarean section (7/17; 41%), and seven (7/17; 41%) decided to terminate the pregnancy.

The gestational age at delivery also differed in the two groups. Three symptomatic women had a miscarriage before 20 weeks of gestation (3/46; 6%), one had a miscarriage between 20 and 25 weeks (1/46; 2.1%), four delivered between 25 and 30 weeks (4/46; 8.6%), 13 delivered between 30 and 35 weeks (13/46; 28.2%), and 15 delivered after 35 weeks (15/46; 32.6%). Three women without symptoms decided to terminate pregnancy before 20 weeks of gestation (3/17; 17%), four terminated pregnancy between 20 and 25 weeks (4/17; 23%), six delivered between 30 and 35 weeks (6/17; 35%), and four delivered after 35 weeks (4/17; 23%).

Termination of pregnancy was performed in one symptomatic woman and in those without symptoms in whom HCC was discovered after AFP analysis. Fifteen women who delivered by cesarean section underwent hepatic surgery at the same time (15/63; 23.8%). Of them, four women received adjuvant chemotherapy and one underwent a transarterial chemoembolization procedure (5/15; 33%).

Nine women (9/63; 14%) were treated during pregnancy: six of them underwent surgery, five between 17 and 28 weeks (5/9; 56%) and one at 6 weeks (1/9; 11%); one received radiofrequency ablation (1/9; 11%); and two women received supportive care and died during hospitalization (2/9; 22%).

Twenty‐six women were treated after delivery (26/63; 41%), they underwent surgery in 17 cases (17/26; 65%), in four cases neoadjuvant chemotherapy was started (4/26; 15%), four of them received a transarterial chemoembolization procedure (4/26; 15%) and one received supportive therapy, dying within 6 months (1/26; 4%).

The maternal and fetal outcomes are described in Table 2. We cannot estimate an overall survival rate of women who had HCC during pregnancy because of the differences in follow‐up data between the reports. The results show that 52% of symptomatic women died within 6 months after diagnosis (23/46), whereas women without symptoms with survival >24 months were 29% (5/17). Notably, no patient with a cirrhotic liver survived more than 12 months.

**TABLE 2:** Maternal and neonatal survival data.

	All (*n* = 63)	Symptomatic (*n* = 46)	Asymptomatic (*n* = 17)
Maternal survival			
Not known	11 (18%)	9 (20%)	2 (12%)
<6 months	26 (41%)	23 (50%)	3 (18%)
6 months < *x* > 12 months	13 (20%)	8 (17%)	5 (29%)
12 months < *x* > 24 months	4 (7%)	2 (4%)	2 (12%)
>24 months	9 (14%)	4 (9%)	5 (29%)
Neonatal survival			
Not known	5 (8%)	4 (9%)	1 (6%)
Survived	38/58 (66%)	28/42 (67%)	10/16 (63%)
Prenatal/perinatal deaths	20/58 (34%)	14/42 (33%)	6/16 (37%)

The cause of death was described in 23 women only: 15 of them (15/23; 65%) died from hepatic insufficiency and multi‐organ failure; five died from lung failure (5/23; 22%); and three from hemorrhagic shock (2/23; 13%).

Thirty‐eight (66%) newborns survived after delivery, 28 (67%) in the symptomatic group and 10 (63%) in women without symptoms (Table 2).

## 4 DISCUSSION

Hepatocellular carcinoma is a rare condition, especially in young women. In this systematic review, we identified 63 cases in pregnant women from 47 case reports and 5 case series, distributed over 70 years. Overall, 42 women (68%) died within 24 months of diagnosis, and we did not have information about 11 women (18%). Fifty percent of symptomatic women died within 6 months after diagnosis. Survival >24 months was 9% in symptomatic and 29% in asymptomatic women. No patient with cirrhotic liver survived more than 12 months. Hepatocellular carcinoma in pregnancy is associated with an increased risk of preterm delivery. Overall neonatal survival rate was 66%.

The strength of our study is the use of raw data to perform statistical analyses accounting for gestational age at diagnosis and delivery, and maternal serum AFP concentrations. The limitations of our systematic review are several: the rarity of this disease in pregnancy, which limited the number of patients analyzed; the presence of only case series and case reports excluded the possibility of performing meta‐analysis and increased the risk of reporting bias; and improvements in diagnosis and treatment over the years, may have affected time to diagnosis, survival rate, and pregnancy outcomes.

Fetal malformation screening including maternal serum AFP assessment led to the diagnosis of HCC in nine women, at a gestational age between 10 and 19 weeks. Seven women of this group decided to terminate the pregnancy in order to receive immediate treatment. Maternal serum AFP concentrations were above the normal range in 28 women, but the test was also performed following the clinical suspicion of HCC and after 18 weeks of pregnancy. Women who received a diagnosis of HCC due to elevated AFP serum levels had the possibility of being treated earlier, which might allow a longer survival (Table 2).

In contrast, the suspicion of HCC based on symptoms was more frequent after 20 weeks of pregnancy, and the most frequent symptom was abdominal pain. In this group, termination of pregnancy was not performed, probably because of gestational age.

From our data, women affected by HCC delivered preterm more frequently than the general population^13^: 31 women delivered before 37 weeks, and 13 women at 37 weeks or later. Details on gestational age at delivery are reported in Table S1. Heterogeneity of the data did not allow us to identify specific patterns for iatrogenic and spontaneous preterm delivery.

Our data suggest that HCC in pregnancy is a rare condition, but the mortality rate and the consequences of the disease are too important to be underestimated. Increased maternal serum AFP, especially if fetal anomalies are excluded, should prompt the obstetrician to exclude the possibility of HCC. However, as second‐trimester maternal serum screening for fetal aneuploidy has been replaced by first‐trimester screening in many parts of the world, the rarity of HCC in pregnancy does not justify maintaining AFP assessment for the sole purpose of HCC detection.

Liver sonography is a safe procedure in pregnancy and should be performed if a suspicion of HCC is raised. The importance of rapid diagnosis is related to the possibility of early treatment, which could improve overall survival. In the presence of risk factors or according to local epidemiology, first‐trimester screening for hepatitis B and/or C virus should be considered. In pregnant women with a diagnosis of HBV infection, liver ultrasound evaluation can exclude both cirrhosis and HCC. Patients with cirrhotic liver had the worst outcomes in our data set, with an overall survival of less than 12 months. Finally, neonatal outcomes were worse than in the general population^14^; this is associated both with gestational age at delivery and maternal conditions at delivery.

## 5 CONCLUSION

Hepatocellular carcinoma in pregnancy is associated with a high risk of maternal and neonatal mortality. Diagnosis in asymptomatic high‐risk women or following abnormal maternal serum AFP screening is associated with better maternal outcomes. On the other hand, liver cirrhosis is associated with a worse outcome and an overall survival less than 12 months.

## AUTHOR CONTRIBUTIONS

FM and FP contributed substantially to the conception and design of this work. FM, IP, FP, and FF planned the analysis, performed statistical analyses, and drafted the manuscript. FM, IP, FP, FF, AF, and FEO contributed substantially to the acquisition of the data. All the authors revised the paper and approved the final version.

## CONFLICT OF INTEREST STATEMENT

The authors have no conflict of interest to declare.

## Supporting information


Table S1.

Table S2.

Table S3.
