# Supplementary material for: Hepatocellular carcinoma in pregnancy: A systematic review
Source: Acta Obstet Gynecol Scand. 2023 Aug 2;103(4):653–9. doi: 10.1111/aogs.14640 (PMC10993335; doi:10.1111/aogs.14640)
Supplement: Supplementary file 1 — Table S1. [file AOGS-103-653-s001.docx]

| Patient number | Publication Year | Age | Ethnicity - Country | Previous disease | Histology | Cirrotic liver | Gestational week at Diagnosis | Clinical presentation | MSAFP Levels/weeks | AFAFP | Maternal survival | | Gestational week at delivery | Neonatal survival | Neonatal weight at delivery (grams) |
| --- | --- | --- | --- | --- | --- | --- | --- | --- | --- | --- | --- | --- | --- | --- | --- |
| 1 | 1957 | 43 | East Asia | N | hepatoma | Y | 30 | Abdominal pain, spontaneous mass rupture | UK |  | < 6 months | 34 | | Y | 1800 |
| 2 | 1964 | 27 | African | N | hepatoma low grade, lever cell type | N | Onset 1 day after delivery | Abdominal Pain | UK |  | > 24 months | 36 | | Y | 4000 |
| 3 | 1964 | 38 | South Asia | Previous blood transfusion | hepatoma of the liver | Y | 36 | Abdominal pain with spontaneous rupture, shock | UK |  | < 6 months | 36 | | Died 2 days neonatally | 3260 |
| 4 | 1974 | 28 | Caucasian | Recurrent HCC | well differentiated HCC | N | 32 | Abdominal Pain | UK |  | < 6 months | UK | | N | 2100 |
| 5 | 1974 | 37 | Latin America | N | poorly differentiated HCC | Y | 25 | Rectal bleeding | 20-25µg/ml/25 | UK | < 6 months | Maternal death | | UK |  |
| 6 | 1977 | 32 | Caucasian | N | HCC | N | Postpartum | Pain and gastrointestinal symptoms | UK |  | < 6 months | UK | | Y |  |
| 7 | 1980 | 29 | African | UK | HCC | UK | 27 | Abdominal mass, jaundice | Normal sieric AFP levels/27 | UK | < 6 months | Stillborn at 27 | | Stillborn | 1056 |
| 8 | 1980 | 31 | Caucasian | N | HCC | N | 19 | Sieric incidental finding | 4600 ng/ml/19 (102 MOM) | UK | UK | TOP at 19 | | N |  |
| 9 | 1981 | 22 | Caucasian | N | HCC | N | 27 | Abdominal Pain | UK |  | UK | 35 | | Y | 2040 |
| 10 | 1981 | 45 | Caucasian | N | HCC | N | 35 | Pain and gastrointestinal symptoms | Normal sieric AFP levels/35 | UK | < 6 months | 37 | | Y |  |
| 11 | 1982 | 33 | African | N | HCC - hepatoma | N | 9 | Abdominal Pain | UK |  | < 6 months | Miscarriage at 9 | | N |  |
| 12 | 1984 | 33 | UK | Hepatic schistosomiasis in HBsAg carrier | HCC | UK | 16 | Hypoglicemic coma | 6300 µg/L/16 | UK | < 6 months | 28 | | N |  |
| 13 | 1986 | 17 | African | N | HCC | UK | 32 | Gastrointestinal symptoms | 90 µg/l/27 | UK | < 6 months | 33 | | Y | 2100 |
| 14 | 1991 | 30 | African | UK | HCC | Y | 32 | Shock and abdominal pain | UK |  | < 6 months | 32 | | Fresh stillborn |  |
| 15 | 1991 | 31 | South Asia | HBsAg carrier | HCC | N | 16 | Sieric incidental finding | 62000 ng/ml/16 | N | 6 months <x< 24 months | TOP at 20 | | N |  |
| 16 | 1991 | 22 | Middle East | N | FLHCC | N | Onset just before pregnancy | Pain and gastrointestinal symptoms | UK |  | 6 months <x< 24 months | 40 | | Y | 3115 |
| 17 | 1991 | 35 | African | HBsAg carrier | poorly differentiated HCC | N | 30 | Abdominal pain and swelling | 100IU/ml/30 | UK | < 6 months | 40 | | Y |  |
| 18 | 1992 | 38 | Caucasian | Chronic hypertension | HCC | N | 32 | Preeclampsia (edema, hypertension, cardiac failure) | UK |  | < 6 months | 33 | | Y | 1900 |
| 19 | 1993 | 21 | East Asia | HBsAg carrier | HCC | Y | Onset 10 days after delivery | Abdominal Pain | 6,6 ng/ml normal/postpartum 10 day | UK | < 6 months | UK | | UK |  |
| 20 | 1993 | 22 | East Asia | HBsAg carrier | single hepatic tumor in cirrotic liver (no histology) | Y | 27 | Abdominal pain and raised AFP | 378,680 ng/ml/17 | N | < 6 months | Maternal death | | N |  |
| 21 | 1993 | 31 | East Asia | HBsAg carrier | HCC | Y | 12 | Sieric incidental finding | 1446 ng/ml/15 | N | < 6 months | 32 | | Y | 2000 |
| 22 | 1993 | 35 | East Asia | HBsAg carrier | HCC | N | 28 | Hepatomegaly | 2951 ng/ml/28 | UK | < 6 months | 30 | | Y | 1580 |
| 23 | 1995 | 28 | East Asia | HBsAg carrier | HCC | N | 40 | Abdominal Pain | 78 ng/ml/40 | UK | 6 months <x< 24 months | 40 | | Y | 2800 |
| 24 | 1995 | 29 | East Asia | HBsAg carrier | HCC | Y | 32 | Sieric incidental finding | 210000 ng/ml/32 | UK | < 6 months | 35 | | Y | 2300 |
| 25 | 1995 | 33 | East Asia | HBsAg carrier | HCC | Y | 38 | Abdominal pain and shock, with mass rupture after delivery | 270 ng/ml/38 | UK | < 6 months | 38 | | Y | 2850 |
| 26 | 1995 | 32 | Caucasian | N | HCC | N | 32 | Abdominal Pain | 400 ng/ml/32 | UK | UK | UK | | UK |  |
| 27 | 1995 | 28 | Caucasian | Alchol intake | HCC | N | 18 | Sieric incidental finding | 3600 ng/ml/18 | UK | 6 months <x< 24 months | TOP at 18 | | N |  |
| 28 | 1995 | 26 | East Asia | HBsAg carrier | HCC G3 | N | 17 | Sieric incidental finding | 34303 ng/ml/17 | N | > 24 months | TOP at 17 | | N | 344 |
| 29 | 1995 | 26 | East Asia | HBsAg carrier | HCC | Y | 16 | Sieric incidental finding | 34303 ng/ml/16 | UK | 6 months <x< 24 months | TOP at 23 | | N |  |
| 30 | 1996 | 22 | Caucasian | N | FLHCC | N | 27 | Abdominal Pain | Normal sieric AFP levels/27 | UK | UK | 29 | | Y |  |
| 31 | 1996 | 26 | East Asia | HBsAg carrier | moderately differentiated HCC | N | 17 | Sieric incidental finding | 6,9 MOM/17 | N | > 24 months | 33 | | Y | 2690 |
| 32 | 1999 | 25 | UK | N | moderately differentiated HCC | N | 19 | Abdominal Pain | 47,3 IU/ml/19 | UK | > 24 months | 34 | | Y | 1804 |
| 33 | 2001 | 33 | East Asia | N | HCC | N | 25 | Abdominal pain with spontaneous mass rupture | 105 ng/ml/25 | UK | > 24 months | 41 | | Y | 2220 |
| 34 | 2001 | 29 | East Asia | Mediastinal B cell lymphoma in HBsAg carrier | undifferentiated HCC | N | 28 | Abdominal Pain | 979200 ng/ml 32 (highest level) | UK | < 6 months | 32 | | Y | 1700 |
| 35 | 2003 | 25 | Caucasian | N | FLHCC | N | Onset 1 day after delivery | Abdominal Pain | 40,5 IU/ml postpartum 7day | UK | 6 months <x< 24 months | 40 | | Y | 3800 |
| 36 | 2004 | 38 | Caucasian | N | moderately differentiated HCC | N | 28 | Abdominal Pain | 401U/l/28 | UK | UK | 30 | | Y | 1400/1490 Twin pregnancy |
| 37 | 2005 | 24 | Latin America | HBsAg carrier | HCC (no histologic data) | N | 19 | Hypoglicemic coma | > 35350 µg/l/19 | UK | UK | UK | | UK |  |
| 38 | 2005 | 28 | East Asia | N | HCC | UK | 29 | Pain and gastrointestinal symptoms | 129,62 ng/ml/29 | UK | > 24 months | 36 | | Y | 1700 |
| 39 | 2005 | 27 | Caucasian | N | FLHCC in FNH | N | 28 | Abdominal Pain | Normal sieric AFP levels/18 | UK | 6 months <x< 24 months | 39 | | Y |  |
| 40 | 2006 | 38 | African | HBsAg carrier | poorly differentiated HCC | N | 12 | Radiological incidental finding | 16738,8 ng/ml/15 | UK | 6 months <x< 24 months | 33 | | Y | 1830 |
| 41 | 2009 | 40 | African | HBsAg carrier | HCC | N | Onset 3 days after delivery | Abdominal Pain | >400 ng/ml/postpartum 3 day | UK | 6 months <x< 24 months | ? | | Y |  |
| 42 | 2010 | 31 | Caucasian | HBsAg carrier | HCC | UK | 26 | Jaundice | 169 ng/ml/26 | UK | 6 months <x< 24 months | 32 | | Y |  |
| 43 | 2012 | 33 | South Asia | N | poorly differentiated HCC | N | 20 | Radiological incidental finding | 295 (ng/ml)/20 | UK | 6 months <x< 24 months | 30 | | Y |  |
| 44 | 2012 | 41 | South Asia | N | HCC | N | 22 | Abdominal pain and jaundice | 90,7 (ng/ml)/22 | UK | < 6 months | 23 | | N |  |
| 45 | 2012 | 30 | East Asia | N | poorly differentiated HCC | N | 25 | Radiological incidental finding | 305 ng/ml/25 | UK | > 24 months | 41 | | Y | 3396 |
| 46 | 2012 | 33 | East Asia | HBsAg carrier | moderately differentiated HCC | N | 28 | Sieric incidental finding | 973 µg/L/28 | UK | < 6 months | 32 | | Y | 1800 |
| 47 | 2014 | 27 | East Asia | HBsAg carrier | HCC | N | 32 | Abdominal Pain | 1000 U/L/32 | UK | < 6 months | UK | | Y | 2900 |
| 48 | 2014 | 24 | East Asia | HBsAg carrier | HCC | N | 26 | Abdominal pain with spontaneous mass rupture | 1000 U/L/26 | UK | 6 months <x< 24 months | Stillborn at 26 | | Stillborn | 1500 |
| 49 | 2014 | 23 | East Asia | HBsAg carrier | HCC | N | 16 | Abdominal Pain | 1000 U/L/16 | UK | < 6 months | Stillborn at 16 | | Stillborn |  |
| 50 | 2014 | 40 | East Asia | HBsAg carrier | HCC | N | 24 | Radiological incidental finding | 1000 U/L/24 | UK | 6 months <x< 24 months | TOP at 24 | | N |  |
| 51 | 2014 | 23 | East Asia | HBsAg carrier | HCC | Y | 38 | Jaundice | 23726 ng/ml/38 | UK | 6 months <x< 24 months | 38 | | Y | 3100 |
| 52 | 2014 | 28 | East Asia | HBsAg carrier | HCC | N | 33 | Radiological incidental finding | 241,41 ng/ml/33 | UK | UK | 38 | | Y |  |
| 53 | 2015 | 30 | Caucasian | Recent hemoangioma bleeding | Combined HCC-CC | N | 30 | Abdominal Pain | UK |  | < 6 months | 31 | | Y | 2200 |
| 54 | 2015 | 30 | African | HIV | HCC (No histologic data) | N | Postpartum | Abdominal pain, fever | 10178 µg/L/postpartum | UK | UK | 32 | | Y | 1525 |
| 55 | 2016 | 36 | African | HBsAg carrier | HCC (no histologic data) | Y | 27 | Abdominal Pain | >50000 U/ml/27 | UK | UK | 30 | | Fresh stillborn | 1200 |
| 56 | 2016 | 32 | South Asia | Hypothyroidism | HCC | Y | 19 | Abdominal pain, intrauterine fetal demise, haemoperitoneum | 64,7 IU/ml/postpartum | UK | UK | 19 | | N |  |
| 57 | 2017 | 26 | South Asia | N | FLHCC | N | 26 | Jaundice | 195,8 IU/ml/26 | UK | 6 months <x< 24 months | 31 | | Y |  |
| 58 | 2018 | 36 | East Asia | HBsAg carrier | HCC | N | 6 | Abdominal pain, vomiting, haemoperitoneum | UK | UK | 6 months <x<24 months | TOP at 7 | | N |  |
| 59 | 2018 | 39 | Caucasian | Recurrent HCC in HHT | well-differentiated HCC | N | 11 | Incidental during follow-up | 150,2 ng/ml/30 (highest level) | UK | 6 months <x< 24 months | 33 | | Y | 2350 |
| 60 | 2018 | 33 | East Asia | HBsAg carrier | moderately differentiated HCC | N | 8 | Incidental during follow-up | 39,2 ng/ml/8 | UK | > 24 months | 37 | | Y |  |
| 61 | 2020 | 27 | African | HBsAg carrier | HCC no histology | Y | 35 | Abdominal Pain | 2232 ng/ml/35 | UK | < 6 months | 35 | | Y | 2900 |
| 62 | 2020 | 36 | East Asia | Recurrent HCC in HBsAg carrier | moderately differentiated HCC | N | 20 | Incidental during follow-up | 12371,5 ng/ml/20 | UK | > 24 months | TOP at 21 | | N |  |
| 63 | 2020 | 31 | Caucasian | N | HCC | N | 40 | Abdominal pain with spontaneous mass bleeding | UK |  | UK | 40 | | UK |  |

Y Yes; N No; HBsAg hepatitis B surface antigen; MSAFP Maternal serum alpha-fetoprotein; AFAFP Amniotic fluid alpha-fetoprotein; UK unknown, HCC Hepatocellular carcinoma; FLHCC Fibrolamellar hepatocarcinoma; CC Cholangiocarcinoma; HIV Human Immunodeficiency Virus; HHT Hereditary hemorrhagic telangiectasia.

**Table S1: The characteristics of each patient**
