# Supplementary material for: Hepatocellular carcinoma in pregnancy: A systematic review
Source: Acta Obstet Gynecol Scand. 2023 Aug 2;103(4):653–9. doi: 10.1111/aogs.14640 (PMC10993335; doi:10.1111/aogs.14640)
Supplement: Supplementary file 2 — Table S2. [file AOGS-103-653-s003.docx]

| **First author** | **Title** | **Type of article** | **Number of patients** | **Does the patient(s) represent(s) the whole experience of the investigator (centre) or is the selection method unclear to the extent that other patients with similar presentation may not have been reported?** | **Was the exposure adequately ascertained?** | **Was the outcome adequately ascertained?** | | **Were other alternative causes that may explain the observation ruled out?** | **Was follow-up long enough for outcomes to occur?** | **Is the case(s) described with sufficient details to allow other investigators to replicate the research or to allow practitioners make inferences related to their own practice?** |
| --- | --- | --- | --- | --- | --- | --- | --- | --- | --- | --- |
| **Gerli** | Mixed hepatocellular carcinoma and cholangiocarcinoma during pregnancy: a case report | CR | 1 | 1 | 1 | 1 | | 1 | 1 | 1 |
| **Russell** | Hepatocellular carcinoma during pregnancy: case report and review of the literature | CR | 1 | 1 | 1 | 1 | | 1 | 1 | 1 |
| **Lau** | Hepatocellular carcinoma during pregnancy and its comparison with other pregnancy-associated malignancies | CS | 5 | 1 | 1 | 1 | | 1 | 1 | 1 |
| **Wang** | Pregnancy associated with primary hepatocellular carcinoma | CS | 3 | 1 | 0 | 0 | | 1 | 1 | 1 |
| **Wembulua** | Hepatitis B-related hepatocellular carcinoma in a 36-year-old pregnant woman: prognosis and management dilemma | CR | 1 | 1 | 0 | 0 | | 1 | 1 | 1 |
| **Maeda** | Hepatic resection for recurrent hepatocellular carcinoma during pregnancy: a case report | CR | 1 | 1 | 1 | 1 | | 1 | 1 | 1 |
| **McCarthy** | Recurrent hepatocellular carcinoma in pregnancy: A case report and literature review | CR | 1 | 1 | 1 | 1 | | 1 | 1 | 1 |
| **Matsuo** | Novel treatment strategy with radiofrequency ablation and surgery for pregnant patients with hepatocellular carcinoma: a case report | CR | 1 | 1 | 1 | 1 | | 1 | 1 | 1 |
| **Ai-Jun Li** | Surgery for pregnancy-associated primary hepatocellular carcinoma: Report of four cases | CS | 4 | 1 | 1 | 1 | | 1 | 1 | 1 |
| **Hua-Wein Chen** | Hepatocellular Carcinoma Presenting with Obstructive Jaundice during Pregnancy | CR | 1 | 1 | 1 | 1 | | 1 | 1 | 1 |
| **Norouzi** | Hepatocellular Carcinoma in Pregnancy withUnusual Presentations | CR | 1 | 1 | 1 | 1 | | 1 | 1 | 1 |
| **Alvarez de la Rosa** | Evolution and management of a hepatocellular carcinoma during pregnancy | CR | 1 | 1 | 1 | 1 | | 1 | 1 | 1 |
| **Garko** | Hepatocellular carcinoma in pregnancy | CR | 1 | 1 | 1 | 1 | | 1 | 1 | 1 |
| **Hsu** | Spontaneous rupture of hepatocellular carcinoma during pregnancy | CR | 1 | 1 | 1 | 1 | | 1 | 1 | 1 |
| **Louie-Johnsun** | Fibrolamellar hepatocellular carcinoma in pregnancy | CR | 1 | 1 | 1 | 1 | | 1 | 1 | 1 |
| **Chen** | Hepatocellular carcinoma in pregnancy | CR | 1 | 1 | 1 | 1 | | 1 | 1 | 1 |
| **Hung** | Surgical resection for hepatocellular carcinoma in pregnancy: a case report | CR | 1 | 1 | 1 | 1 | | 1 | 1 | 1 |
| **Cedron Cheng** | [Severe hypoglycemia as first manifestation hepatocarcinoma in pregnancy: case report] | CR | 1 | 1 | 0 | 0 | | 1 | 0 | 1 |
| **Brucker** | Hepatocellular carcinoma in pregnancy | CR | 1 | 1 | 1 | 1 | | 1 | 0 | 1 |
| **To** | Primary liver carcinoma complicating pregnancy | CR | 1 | 1 | 1 | 1 | | 1 | 1 | 1 |
| **Ngawuchu** | Rupture of a nodule of hepatocellular carcinoma simulating uterine rupture in late pregnancy | CR | 1 | 1 | 1 | 1 | | 1 | 1 | 1 |
| **Goldberg** | A case of hepatocellular carcinoma in pregnancy detected by routine screening of maternal alpha-feto-protein | CR | 1 | 1 | 1 | 1 | | 1 | 1 | 1 |
| **Egwuatu** | Primary hepatocarcinoma in pregnancy | CR | 1 | 1 | 1 | 1 | | 1 | 1 | 1 |
| **HW Chen** | Synchronous right hepatectomy and cesarean section in a pregnant lady with hepatocellular carcinoma | CR | 1 | 1 | 1 | 1 | | 1 | 1 | 1 |
| **Scioscia** | Spontaneous bleeding of hepatocellular carcinoma during pregnancy | CR | 1 | 1 | 1 | 1 | | 1 | 0 | 1 |
| **Vishnu** | Fibrolamellar variant of hepatocellular carcinoma presenting during pregnancy: management dilemmas | CR | 1 | 1 | 1 | 1 | | 1 | 1 | 1 |
| **Mnyani** | Delayed presentation and diagnosis of metastatic hepatocellular carcinoma in pregnancy | CR | 1 | 1 | 0 | 0 | | 1 | 0 | 1 |
| **Au** | Aggressive hepatocellular carcinoma complicating pregnancy after autologous bone marrow transplantation for non-Hodgkin's lymphoma | CR | 1 | 1 | 1 | 1 | | 1 | 1 | 1 |
| **Gisi** | Hepatocellular carcinoma in pregnancy. A case report | CR | 1 | 1 | 1 | 1 | | 1 | 1 | 1 |
| **Hsieh** | Term delivery after hepatocellular carcinoma resection in previous pregnancy | CR | 1 | 1 | 1 | 1 | | 1 | 1 | 1 |
| **Jeng** | Hepatocellular carcinoma in a pregnant woman detected by routine screening of maternal alpha-fetoprotein | CR | 1 | 1 | 1 | 1 | | 1 | 1 | 1 |
| **Seaward** | Primary hepatocellular carcinoma in pregnancy. A case report | CR | 1 | 1 | 1 | 1 | | 1 | 1 | 1 |
| **Goncalves** | Hepatocellular carcinoma HBsAg positive in pregnancy | CR | 1 | 1 | 1 | 1 | | 1 | 1 | 1 |
| **Dudley** | Hepatocellular carcinoma associated with oral contraceptive use and pregnancy | CR | 1 | 1 | 1 | 1 | | 1 | 1 | 1 |
| **Zimmer** | An unusual cause of jaundice in pregnancy | CR | 1 | 1 | 1 | 1 | | 1 | 1 | 1 |
| **Nam** | A case of the hepatocellular carcinoma during the pregnancy and metastasis to the left atrium | CR | 1 | 1 | 1 | 1 | | 1 | 1 | 1 |
| **Pajor** | Pregnancy in primary liver carcinoma | CR | 1 | 1 | 1 | 1 | | 1 | 1 | 1 |
| **Pietsch** | Liver tumours in pregnancy, following previous intake of hormonal contraceptives (author's transl) | CS | 1 | 1 | 1 | 1 | | 1 | 0 | 1 |
| **Balazs** | Primary carcinoma of the liver and long-term administration of oral contraceptives followed by pregnancy (author's transl) | CR | 1 | 1 | 1 | 1 | | 1 | 1 | 1 |
| **Kim** | SILENT PRIMARY CARCINOMA OF THE LIVER COMPLICATING PREGNANCY: REPORT OF A CASE | CR | 1 | 1 | 1 | 1 | | 1 | 1 | 1 |
| **Yen** |  | CR | 1 | 1 | 1 | | 1 | 1 | 1 | 1 |
| **Roddie** | Haemorrhage from primary carcinoma of the liver complicating pregnancy | CR | 1 | 1 | 1 | 1 | | 1 | 1 | 1 |
| **Awuku Y.A.** | Management dilemma of hepatocellular carcinoma in pregnancy: A case report | CR | 1 | 1 | 0 | 0 | | 1 | 0 | 1 |
| **Kutty D.N.** | A rare case of haemoperitoneum in pregnancy | CR | 1 | 1 | 1 | 1 | | 1 | 0 | 1 |
| **Janssen P.** | Hepatocellular carcinoma during pregnancy after IVF | CR | 1 | 1 | 1 | 1 | | 1 | 0 | 1 |
| **Entezami M** | Hepatocellular carcinoma as a rare cause of an excessive increase in α- fetoprotein during pregnancy | CR | 1 | 1 | 1 | 1 | | 1 | 1 | 1 |
| **Purtilo** | Primary hepatic malignancy in pregnant women | CS | 2 | 1 | 1 | 1 | | 1 | 1 | 1 |
| **Haddow** | Maternal Hepatoma detected during serum AFP screening | CR | 1 | 1 | 1 | 1 | | 1 | 0 | 1 |
| **Christensen** | A case of hepatoma in pregnancy associated with earlier oral contraception | CR | 1 | 1 | 1 | 1 | | 1 | 1 | 1 |
| **Kroll** | Fibrolamellar carcinoma of the liver in pregnancy. A case report. | CR | 1 | 1 | 1 | 1 | | 1 | 1 | 1 |
| **Imkie** | Fibrolamellar hepatocellular carcinoma arising in a background of focal nodular hyperplasia: a report of 2 cases | CR | 1 | 1 | 1 | 1 | | 1 | 1 | 1 |
| **Ozoh** | Hepatoma in pregnancy | CR | 1 | 1 | 1 | 1 | | 1 | 1 | 1 |
| **Lee** | Ruptured hepatocellular carcinoma disguising as heterotopic pregnancy | CR | 1 | 1 | 1 | 1 | | 1 | 1 | 1 |

**Table S2. The assessment of the quality and risk of bias of each article.**
