# Supplementary material for: Hepatocellular carcinoma in pregnancy: A systematic review
Source: Acta Obstet Gynecol Scand. 2023 Aug 2;103(4):653–9. doi: 10.1111/aogs.14640 (PMC10993335; doi:10.1111/aogs.14640)
Supplement: Supplementary file 3 — Table S3. [file AOGS-103-653-s002.docx]

| **First author** | **Title** | **AMA Citations** | **Incomplete data** | **Not pertinent** | **Not relevant** | **Not meet criteria** | **Not retrieved** |
| --- | --- | --- | --- | --- | --- | --- | --- |
| Nerad | Pregnancy following left-sided lobectomy of the liver during recurring hepatoma | Nerad V, Skaunic V. Tĕhotenství po levostranné lobektomii jater v průbĕhu recidivujícího hepatomu [Pregnancy following left-sided lobectomy of the liver during recurring hepatoma]. Cas Lek Cesk. 1969 Feb 21;108(8):228-9. Czech. PMID: 4305174. |  |  |  |  | x |
| Yau J.-H. | Hepatocellular carcinoma during pregnancy: A case report | Yau, J.-H & Chilcote, D. & Eppes, Carey. Hepatocellular carcinoma during pregnancy: A case report. Journal of Reproductive Medicine. 2019 64 67-70. |  |  |  |  | x |
| Naidoo T.D | Hepatocellular carcinoma in pregnancy: A case report and evaluation of current management trends | Naidoo T.D., Govender L.,Green-Thompson,R. R. Hepatocellular carcinoma in pregnancy: A case report and evaluation of current management trends. Obstetrics and Gynaecology (2019) https://hdl.handle.net/10520/EJC-17aa48fc22 |  |  |  |  | x |
| Chu J.-S | Concomitant digestive malignancy in pregnancy | Chu J.-S, Yang K.-C., Hsu T.-C., Kao C.-R, Chou S.-Y, Shih S.-C. Concomitant digestive malignancy in pregnancy. Chinese Journal of Perinatology 1993 10:1 |  |  |  |  | x |
| Francis T.I | Hepatocellular carcinoma in pregnancy | Francis TI, Smith JA, Hendrickse JP. Hepatocellular carcinoma in pregnancy. East Afr Med J. 1974 Nov;51(11 SPEC NO):789-800. PMID: 4376479. |  |  |  |  | x |
| Dahan | Fibrolamellar hepatic carcinoma with a presentation similar to that of septic pregnancy. A case report | Dahan MH, Kastell P. Fibrolamellar hepatic carcinoma with a presentation similar to that of septic pregnancy. A case report. J Reprod Med. 2002 Jan;47(1):47-9. PMID: 11838311. |  |  |  |  | x |
| Cho | Two cases of the hepatocellular carcinoma in pregnancy | Cho JS, Han KH, Lee WJ, et al. Two Cases of the Hepatocellular Cancinoma in Pregnancy. J Korean Surg Soc. 1997;52(4):598-605. |  |  |  |  | x |
| Choi | A case of hepatocellular carcinoma in a pregnant patient in twenties | Choi KK, Hong YJ, Choi SB, Yi NJ, Hwang S, Park YN, et al. A case of hepatocellular carcinoma in a pregnant patient in twenties. J Korean Liver Cancer Study Group. 2009;9:76–81 |  |  |  |  | x |
| Kim | Three cases of primary hepatocellular carcinoma first detected during pregnancy | Kim SI, Han SY, Yoon HD et al. 3 Cases of Primary Hepatocellular Carcinoma First Detected During Pregnancy. Korean J Obstet Gynecol 2000 43;12 2323-2327 http://www.ogscience.org/journal/view.php?number=3956 |  |  |  |  | x |
| Kim | A case of hepatocellular carcinoma associated with oral contraceptives in pregnancy | Kim DJ, Lee SK, Oh SY, Kang YS, Kim SS, Park JJ, et al. A case of hepatocellular carcinoma associated with oral contraceptives in pregnancy. Korean J Gastroenterol. 1997;30(1):126–30. |  |  |  |  | x |
| Gemer | Pregnancy in a patient with fibrolamellar hepatocellular carcinoma | Gemer O, Segal S, Zohav E. Pregnancy in a patient with fibrolamellar hepatocellular carcinoma. Arch Gynecol Obstet. 1994;255(4):211-2. doi: 10.1007/BF02335087. PMID: 7695368. |  |  |  | x |  |
| Check | Uncomplicated pregnancy following oral contraceptive-induced liver hepatoma | Check JH, King LC, Rakoff AE. Uncomplicated pregnancy following oral contraceptive-induced liver hepatoma. Obstet Gynecol. 1978 Jul;52(1 Suppl):28S-29S. PMID: 210433. |  |  |  | X |  |
| Qasrawi | Intrahepatic Cholangiocarcinoma Masquerading as Acute Fatty Liver of Pregnancy: A Case Report and Review of the Literature | Qasrawi A, Abughanimeh O, Abu Ghanimeh M, Arora-Elder S, Yousef O, Tamimi T. Intrahepatic Cholangiocarcinoma Masquerading as Acute Fatty Liver of Pregnancy: A Case Report and Review of the Literature. Case Reports Hepatol. 2018 Feb 26;2018:6939747. doi: 10.1155/2018/6939747. PMID: 29682369; PMCID: PMC5846373. |  |  |  | x |  |
| Pritze | Pregnancy after successful therapy of HBV-associated hepatocellular cancer--a case report | Pritze W, Ebert A, Weitzel HK. Schwangerschaften nach erfolgreicher Therapie eines HBV-assoziierten hepatozellulären Carcinomas (HCC)--eine Kasuistik [Pregnancy after successful therapy of HBV-associated hepatocellular cancer--a case report]. Zentralbl Gynakol. 1992;114(11):560-3. German. PMID: 1336289. |  |  |  | x |  |
| Francis | Recurrent Cholangiocarcinoma in Pregnancy: A Case Report | Francis AP, Chang M, Dolin CD, Chervenak J, Cardonick E. Recurrent Cholangiocarcinoma in Pregnancy: A Case Report. AJP Rep. 2018 Oct;8(4):e261-e263. doi: 10.1055/s-0038-1675376. Epub 2018 Nov 12. PMID: 30425881; PMCID: PMC6232044. |  |  |  | x |  |
| Su | Prognostic Significance of Pregnancy Zone Protein and Its Correlation with Immune Infiltrates in Hepatocellular Carcinoma | Su L, Zhang G, Kong X. Prognostic Significance of Pregnancy Zone Protein and Its Correlation with Immune Infiltrates in Hepatocellular Carcinoma. Cancer Manag Res. 2020 Oct 9;12:9883-9891. doi: 10.2147/CMAR.S269215. PMID: 33116846; PMCID: PMC7553665. |  | x |  |  |  |
| Cho | Expression of Pregnancy Up-regulated Non-ubiquitous Calmodulin Kinase (PNCK) in Hepatocellular Carcinoma | Cho YA, Choi S, Park S, Park CK, Ha SY. Expression of Pregnancy Up-regulated Non-ubiquitous Calmodulin Kinase (PNCK) in Hepatocellular Carcinoma. Cancer Genomics Proteomics. 2020 Nov-Dec;17(6):747-755. doi: 10.21873/cgp.20229. PMID: 33099476; PMCID: PMC7675661. |  | x |  |  |  |
| Cobey FC | A review of liver masses in pregnancy and a proposed algorithm for their diagnosis and management | Cobey FC, Salem RR. A review of liver masses in pregnancy and a proposed algorithm for their diagnosis and management. Am J Surg. 2004 Feb;187(2):181-91. doi: 10.1016/j.amjsurg.2003.11.016. PMID: 14769302. |  |  | x |  |  |
| Ahmed | Liver diseases in pregnancy: Diseases not unique to pregnancy | Ahmed KT, Almashhrawi AA, Rahman RN, Hammoud GM, Ibdah JA. Liver diseases in pregnancy: diseases unique to pregnancy. World J Gastroenterol. 2013 Nov 21;19(43):7639-46. doi: 10.3748/wjg.v19.i43.7639. PMID: 24282353; PMCID: PMC3837262. |  |  | x |  |  |
| Benson | The liver in pregnancy | Benson A., Oren R., 54 - The Liver in Pregnancy, Editor(s): Arun J. Sanyal, Thomas D. Boyer, Keith D. Lindor, Norah A. Terrault, Zakim and Boyer's Hepatology (Seventh Edition), Elsevier, 2018, Pages 817-836.e5, ISBN 9780323375917, https://doi.org/10.1016/B978-0-323-37591-7.00054-9. |  |  | x |  |  |
| Lee | Pregnancy facilitates maternal liver regeneration after partial hepatectomy | Lee J, Garcia V, Nambiar SM, Jiang H, Dai G. Pregnancy facilitates maternal liver regeneration after partial hepatectomy. Am J Physiol Gastrointest Liver Physiol. 2020 Apr 1;318(4):G772-G780. doi: 10.1152/ajpgi.00125.2019. Epub 2020 Jan 31. PMID: 32003603; PMCID: PMC7191459. |  | x |  |  |  |
| Oana A.-M. | Prevalence of chronic viral hepatitis b non D and C in pregnancy - Prospective study | Oana A.-M, Anton C., Onofrei R.E., Anton E. Prevalence of chronic viral hepatitis b non D and C in pregnancy - Prospective study. Journal of Gastrointestinal and Liver Diseases 2019; 28 Supplement 2 (74). |  | x |  |  |  |
| Jabbour | Major hepatobiliary surgery during pregnancy: safety and timing | Jabbour N, Brenner M, Gagandeep S, Lin A, Genyk Y, Selby R, Mateo R. Major hepatobiliary surgery during pregnancy: safety and timing. Am Surg. 2005 Apr;71(4):354-8. PMID: 15943413. |  |  | x |  |  |
| Rodriguez | Hepatocellular carcinoma during pregnancy and its comparison with other pregnancy-associated malignancies | Rodriguez JM. Hepatocellular carcinoma during pregnancy and its comparison with other pregnancy-associated malignancies. Cancer. 1995 Nov 1;76(9):1678-9. doi: 10.1002/1097-0142(19951101)76:9<1678::aid-cncr2820760927>3.0.co;2-3. PMID: 8635075. |  |  | x |  |  |
| De Flora | Metabolic activation of a cigarette smoke condensate by woodchuck liver, as related to sex, pregnancy, hepatitis virus infection and primary hepatocellular carcinoma | De Flora S, Izzotti A, D'Agostini F, Balansky R, Camoirano A. Metabolic activation of a cigarette smoke condensate by woodchuck liver, as related to sex, pregnancy, hepatitis virus infection and primary hepatocellular carcinoma. Mutat Res. 1994 Aug;324(4):153-8. doi: 10.1016/0165-7992(94)90013-2. PMID: 7519739. |  | x |  |  |  |
| Wang | Congenital biliary atresia in an infant born to hepatitis B mother treated with telbivudine before and during pregnancy | Wang R, Fang S, Sun Q, Zhou YH. Congenital biliary atresia in an infant born to hepatitis B mother treated with telbivudine before and during pregnancy. Dig Liver Dis. 2018 Oct;50(10):1097-1098. doi: 10.1016/j.dld.2018.07.030. Epub 2018 Aug 1. PMID: 30115574. |  | x |  |  |  |
| Chen | Images of the month. Painful abdominal distension during pregnancy | Chen PH, Chang CY, Tsai YJ, Mao SP, Liang HH. Images of the month. Painful abdominal distension during pregnancy. Am J Gastroenterol. 2012 Sep;107(9):1296. doi: 10.1038/ajg.2012.283. PMID: 22951871. | x |  |  |  |  |
| Mizejewski | Cancer during pregnancy: what is the role of maternal serum and placental biomarkers? A review and commentary | Mizejewski GJ. Cancer during pregnancy: what is the role of maternal serum and placental biomarkers? A review and commentary. Tumori. 2014 Nov-Dec;100(6):581-9. doi: 10.1700/1778.19254. PMID: 25688490. |  |  | x |  |  |
| Jonas | Hepatitis B and pregnancy: an underestimated issue | Jonas MM. Hepatitis B and pregnancy: an underestimated issue. Liver Int. 2009 Jan;29 Suppl 1:133-9. doi: 10.1111/j.1478-3231.2008.01933.x. PMID: 19207977. |  | x |  |  |  |
| Jacobson | Hormones of pregnancy, alpha-feto protein, and reduction of breast cancer risk | Jacobson HI, Lemanski N, Narendran A, Agarwal A, Bennett JA, Andersen TT. Hormones of pregnancy, alpha-feto protein, and reduction of breast cancer risk. Adv Exp Med Biol. 2008;617:477-84. doi: 10.1007/978-0-387-69080-3_47. PMID: 18497072. |  | x |  |  |  |
| Pauli Magnus | Sequence analysis of bile salt export pump (ABCB11) and multidrug resistance p-glycoprotein 3 (ABCB4, MDR3) in patients with intrahepatic cholestasis of pregnancy | Pauli-Magnus C, Lang T, Meier Y, Zodan-Marin T, Jung D, Breymann C, Zimmermann R, Kenngott S, Beuers U, Reichel C, Kerb R, Penger A, Meier PJ, Kullak-Ublick GA. Sequence analysis of bile salt export pump (ABCB11) and multidrug resistance p-glycoprotein 3 (ABCB4, MDR3) in patients with intrahepatic cholestasis of pregnancy. Pharmacogenetics. 2004 Feb;14(2):91-102. doi: 10.1097/00008571-200402000-00003. PMID: 15077010. |  | x |  |  |  |
| Maymon | Primary hepatoid carcinoma of ovary in pregnancy | Maymon E, Piura B, Mazor M, Bashiri A, Silberstein T, Yanai-Inbar I. Primary hepatoid carcinoma of ovary in pregnancy. Am J Obstet Gynecol. 1998 Sep;179(3 Pt 1):820-2. doi: 10.1016/s0002-9378(98)70092-4. PMID: 9757999. |  | x |  |  |  |
| Chen | Immunoelectrophoretic differentiation of alpha-fetoprotein in disorders with elevated serum alpha-fetoprotein levels or during pregnancy | Chen RJ, Chen CK, Chang DY, Chow SN, Huang SC, Hsieh CY, Lin MC, Hsu HC. Immunoelectrophoretic differentiation of alpha-fetoprotein in disorders with elevated serum alpha-fetoprotein levels or during pregnancy. Acta Oncol. 1995;34(7):931-5. doi: 10.3109/02841869509127207. PMID: 7492383. |  | x |  |  |  |
| Wilhelm | Oral contraceptive-induced liver tumors and pregnancy | Wilhelm C, Quaas L, Zahradnik HP, Breckwoldt M. Durch orale Kontrazeptiva induzierte Lebertumoren und Schwangerschaft [Oral contraceptive-induced liver tumors and pregnancy]. Geburtshilfe Frauenheilkd. 1990 Oct;50(10):806-9. German. doi: 10.1055/s-2008-1026368. PMID: 1962751. |  | x |  |  |  |
| Monks | Spontaneous rupture of an hepatic adenoma in pregnancy with survival of mother and fetus | Monks PL, Fryar BG, Biggs WW. Spontaneous rupture of an hepatic adenoma in pregnancy with survival of mother and fetus. Aust N Z J Obstet Gynaecol. 1986 May;26(2):155-7. doi: 10.1111/j.1479-828x.1986.tb01555.x. PMID: 3021108. |  | x |  |  |  |
| Barnes | Successful pregnancy following partial hepatectomy for removal of hepatocellular adenomas | Barnes AD, Harder E, Toot PJ. Successful pregnancy following partial hepatectomy for removal of hepatocellular adenomas. Am J Obstet Gynecol. 1984 Dec 15;150(8):998. doi: 10.1016/0002-9378(84)90397-1. PMID: 6095667. |  | x |  |  |  |
| Pilotti | Hepatoblastoma in an infant after Estroprogestational intake by the mother during pregnancy | Pilotti G, Bosco M, Leo D, Ricci C, Suria G. Epatoblastoma in lattante dopo assunzione di estroprogestinici da parte della madre in gravidanza [Hepatoblastoma in an infant after Estroprogestational intake by the mother during pregnancy]. Pediatr Med Chir. 1984 Mar-Apr;6(2):323-5. Italian. PMID: 6099553. |  | x |  |  |  |
| Nagai | The fatty acid levels of rat alpha-fetoprotein derived from fetuses, pregnancy and hepatoma sera | Nagai M, Becker JL, Deutsch HF. The fatty acid levels of rat alpha-fetoprotein derived from fetuses, pregnancy and hepatoma sera. Oncodev Biol Med. 1982;3(5-6):343-50. PMID: 6183647. |  | x |  |  |  |
| Lundgren | Cell lines with spontaneous secretion of pregnancy-associated alpha 2-globulin | Lundgren E, Damber MG, Roos G, von Schoultz B, Stigbrand T, Nilsson K, Alexander JJ. Cell lines with spontaneous secretion of pregnancy-associated alpha 2-globulin. Int J Cancer. 1979 Jul 15;24(1):45-8. doi: 10.1002/ijc.2910240108. PMID: 90021. |  | x |  |  |  |
| Hibbard | Spontaneous rupture of the liver in pregnancy: a report of eight cases | Hibbard LT. Spontaneous rupture of the liver in pregnancy: a report of eight cases. Am J Obstet Gynecol. 1976 Oct 1;126(3):334-8. doi: 10.1016/0002-9378(76)90545-7. PMID: 183505. |  |  | x |  |  |
| Sell | Tissue sites of alpha fetoprotein synthesis by the rat during pregnancy and hepatoma growth | Sell S, Skelly H. Tissue sites of alpha fetoprotein synthesis by the rat during pregnancy and hepatoma growth. J Natl Cancer Inst. 1976 Mar;56(3):645-8. doi: 10.1093/jnci/56.3.645. PMID: 56449. |  | x |  |  |  |
| Nomura | Tumor induction in the progeny of mice receiving 4-nitroquinoline 1-oxide and N-methyl-N-nitrosourethan during pregnancy or lactation | Nomura T, Okamoto E, Tateishi N, Kimura S, Isa Y. Tumor induction in the progeny of mice receiving 4-nitroquinoline 1-oxide and N-methyl-N-nitrosourethan during pregnancy or lactation. Cancer Res. 1974 Dec;34(12):3373-8. PMID: 4371957. |  | x |  |  |  |
| Heinonen | Immunization during pregnancy against poliomyelitis and influenza in relation to childhood malignancy | Heinonen OP, Shapiro S, Monson RR, Hartz SC, Rosenberg L, Slone D. Immunization during pregnancy against poliomyelitis and influenza in relation to childhood malignancy. Int J Epidemiol. 1973 Autumn;2(3):229-35. doi: 10.1093/ije/2.3.229. PMID: 4359832. |  | x |  |  |  |
| Purves | Serum alpha-feto-protein. VI. The radio-immunoassay evidence for the presence of AFP in the serum of normal people and during pregnancy | Purves LR, Purves M. Serum alpha-feto-protein. VI. The radio-immunoassay evidence for the presence of AFP in the serum of normal people and during pregnancy. S Afr Med J. 1972 Sep 9;46(36):1290-7. PMID: 4118727. |  | x |  |  |  |
| Tashiro | Effect of pregnancy on growth of rat ascites tumors | Tashiro T, Sakurai Y. Effect of pregnancy on growth of rat ascites tumors. Gan. 1967 Feb;58(1):97-100. PMID: 4293128. |  | x |  |  |  |
| Lao | Implications of abnormal liver function in pregnancy and non-alcoholic fatty liver disease | Lao TT. Implications of abnormal liver function in pregnancy and non-alcoholic fatty liver disease. Best Pract Res Clin Obstet Gynaecol. 2020 Oct;68:2-11. doi: 10.1016/j.bpobgyn.2020.02.011. Epub 2020 Mar 7. PMID: 32312689. |  | x |  |  |  |
| Jiang | Plasma DNA end-motif profiling as a fragmentomic marker in cancer, pregnancy, and transplantation | Jiang P, Sun K, Peng W, Cheng SH, Ni M, Yeung PC, Heung MMS, Xie T, Shang H, Zhou Z, Chan RWY, Wong J, Wong VWS, Poon LC, Leung TY, Lam WKJ, Chan JYK, Chan HLY, Chan KCA, Chiu RWK, Lo YMD. Plasma DNA End-Motif Profiling as a Fragmentomic Marker in Cancer, Pregnancy, and Transplantation. Cancer Discov. 2020 May;10(5):664-673. doi: 10.1158/2159-8290.CD-19-0622. Epub 2020 Feb 28. PMID: 32111602. |  | x |  |  |  |
| Adam S. | Nitisinone therapy during tyrosinaemia type 1 pregnancy | Adam S.; Robinson P.; Galloway P.; Richmond J.; Turner C. Nitisinone therapy during tyrosinaemia type 1 pregnancy. Journal of Inherited Metabolic Disease (2019) 42 Supplement 1 (128-129) |  | x |  |  |  |
| Hagstrom H. | Body mass index in early pregnancy and future risk of severe liver disease: a population-based cohort study | Hagström H, Höijer J, Andreasson A, Bottai M, Johansson K, Ludvigsson JF, Stephansson O. Body mass index in early pregnancy and future risk of severe liver disease: a population-based cohort study. Aliment Pharmacol Ther. 2019 Mar;49(6):789-796. doi: 10.1111/apt.15162. Epub 2019 Feb 3. PMID: 30714185. |  | x |  |  |  |
| Freese K. | Pregnancy-associated plasma protein-A as potential novel target and serum marker for hepatic fibrosis | Freese K., Thasler W.E., Bosserhoff A.K., Hellerbrand C. Pregnancy-associated plasma protein-A as potential novel target and serum marker for hepatic fibrosis. Zeitschrift fur Gastroenterologie. 2019 57:1 (e12-e13) |  | x |  |  |  |
| Zeng | Effectiveness of tenofovir or telbivudine in preventing HBV vertical transmission for pregnancy | Zeng J, Zheng C, Li H. Effectiveness of tenofovir or telbivudine in preventing HBV vertical transmission for pregnancy. Medicine (Baltimore). 2019 Apr;98(14):e15092. doi: 10.1097/MD.0000000000015092. PMID: 30946367; PMCID: PMC6455986. |  | x |  |  |  |
| Aslam | Management of chronic hepatitis B during pregnancy | Aslam A, Campoverde Reyes KJ, Malladi VR, Ishtiaq R, Lau DTY. Management of chronic hepatitis B during pregnancy. Gastroenterol Rep (Oxf). 2018 Nov;6(4):257-262. doi: 10.1093/gastro/goy025. Epub 2018 Jul 24. PMID: 30430013; PMCID: PMC6225824. |  | x |  |  |  |
| Kim F. | Increased expression of pregnancy-associated plasma protein-A in activated hepatic stellate cells is a potential therapeutic target and serum marker of hepatic fibrosis | Kim F., Hellerbrand C. Increased expression of pregnancy-associated plasma protein-A in activated hepatic stellate cells is a potential therapeutic target and serum marker of hepatic fibrosis. Journal of Hepatology 2018. 68 Supplement 1 |  | x |  |  |  |
| Freese K. | Pregnancy-Associated plasma protein-A is increased in hepatic fibrosis and affects proliferation and activation of hepatic stellate cells | Freese K., Hellerbrand C. Pregnancy-Associated plasma protein-A is increased in hepatic fibrosis and affects proliferation and activation of hepatic stellate cells. Zeitschrift fur Gastroenterologie. 2018 56:1 |  | x |  |  |  |
| Nguyen M.H. | Management of chronic hepatitis B during pregnancy | Nguyen M.H. Management of chronic hepatitis B during pregnancy. Topics in Antiviral Medicine 2017 25:1 Supplement 1 |  | x |  |  |  |
| Munoz-Gamez J.A. | Hepatitis C during pregnancy, vertical transmission and new treatment possibilities | Muñoz-Gámez JA, Salmerón J, Ruiz-Extremera Á. Hepatitis C durante la gestación, transmisión vertical y nuevas posibilidades de tratamiento [Hepatitis C during pregnancy, vertical transmission and new treatment possibilities]. Med Clin (Barc). 2016 Dec 2;147(11):499-505. Spanish. doi: 10.1016/j.medcli.2016.04.003. Epub 2016 May 18. PMID: 27209226. |  | x |  |  |  |
| Lozano-Masdemont B. | Urticarial exanthema due to hepatitis B in a pregnant woman, mimicking a polymorphic eruption of pregnancy | Lozano-Masdemont B, Gómez-Recuero-Muñoz L, Pulido-Pérez A, Molina-López I, Suárez-Fernández R. Urticarial exanthema due to hepatitis B in a pregnant woman, mimicking a polymorphic eruption of pregnancy. Clin Exp Dermatol. 2016 Dec;41(8):896-898. doi: 10.1111/ced.12936. Epub 2016 Oct 20. PMID: 27761922. |  | x |  |  |  |
| Freese K. | Increased expression of pregnancy-associated plasma protein-A (PAPP-A) in hepatic stellate cells correlates with hepatic fibrosis and can be detected in the serum of patients with liver disease | Freese K., Thasler W.E., Hellerbrand C. Increased expression of pregnancy-associated plasma protein-A (PAPP-A) in hepatic stellate cells correlates with hepatic fibrosis and can be detected in the serum of patients with liver disease. Zeitschrift fur Gastroenterologie. 2016 54:12 |  | x |  |  |  |
| Dogra S. | Rose-colored urine, not glasses: A rare etiology of chronic pain in pregnancy | Dogra S., Wong J., Boka K. Rose-colored urine, not glasses: A rare etiology of chronic pain in pregnancy Chest 2016 150:4 Supplement 1 |  | x |  |  |  |
| Fouquet A. | Hepatitis B and pregnancy. Part 1. Thirteen practical issues in antenatal period | Fouquet A, Jambon AC, Canva V, Bocket-Mouton L, Gottrand F, Subtil D. Hépatite B et grossesse. Partie 1. Treize questions pratiques en période anténatale [Hepatitis B and pregnancy. Part 1. Thirteen practical issues in antenatal period]. J Gynecol Obstet Biol Reprod (Paris). 2016 Jun;45(6):531-9. French. doi: 10.1016/j.jgyn.2016.02.004. Epub 2016 Mar 8. PMID: 26964700. |  | x |  |  |  |
| Sangkomkamhang U.S | Hepatitis B vaccination during pregnancy for preventing infant infection | Sangkomkamhang US, Lumbiganon P, Laopaiboon M. Hepatitis B vaccination during pregnancy for preventing infant infection. Cochrane Database Syst Rev. 2014 Nov 11;2014(11):CD007879. doi: 10.1002/14651858.CD007879.pub3. PMID: 25385500; PMCID: PMC7185858. |  | x |  |  |  |
| Linton A. | Raised ferritin in pregnancy-so what? | Linton A., Bhatia K., Rokicka M. Raised ferritin in pregnancy-so what? BJOG: An International Journal of Obstetrics and Gynaecology 2014 121 |  | x |  |  |  |
| Park J.S. | Current recommendations of managing HBV infection in preconception or pregnancy | Park JS, Pan C. Current recommendations of managing HBV infection in preconception or pregnancy. Front Med. 2014 Jun;8(2):158-65. doi: 10.1007/s11684-014-0340-4. Epub 2014 May 29. PMID: 24871444. |  | x |  |  |  |
| Wong F. | Hepatitis B in pregnancy: A concise review of neonatal vertical transmission and antiviral prophylaxis | Wong F, Pai R, Van Schalkwyk J, Yoshida EM. Hepatitis B in pregnancy: a concise review of neonatal vertical transmission and antiviral prophylaxis. Ann Hepatol. 2014 Mar-Apr;13(2):187-95. PMID: 24552860. |  | x |  |  |  |
| Patton H | Management of hepatitis B during pregnancy | Patton H, Tran TT. Management of hepatitis B during pregnancy. Nat Rev Gastroenterol Hepatol. 2014 Jul;11(7):402-9. doi: 10.1038/nrgastro.2014.30. Epub 2014 Apr 1. PMID: 24686270; PMCID: PMC6658169. |  | x |  |  |  |
| Qirko R. | Evaluation of hepatitis B infection in pregnancy and fetal outcome | Qirko R., Goga M., Leli S., Shahinaj R., Haxhihyseni A., Kulenica E. Evaluation of hepatitis B infection in pregnancy and fetal outcome. Journal of Perinatal Medicine 2013 41 |  | x |  |  |  |
| Stewart R.D | Hepatitis B vaccination in pregnancy in the United States | Stewart RD, Sheffield JS. Hepatitis B Vaccination in Pregnancy in the United States. Vaccines (Basel). 2013 May 8;1(2):167-73. doi: 10.3390/vaccines1020167. PMID: 26343965; PMCID: PMC4515586. |  | x |  |  |  |
| Cheung K.W | Towards complete eradication of hepatitis B infection from perinatal transmission: Review of the mechanisms of in utero infection and the use of antiviral treatment during pregnancy | Cheung KW, Seto MT, Wong SF. Towards complete eradication of hepatitis B infection from perinatal transmission: review of the mechanisms of in utero infection and the use of antiviral treatment during pregnancy. Eur J Obstet Gynecol Reprod Biol. 2013 Jul;169(1):17-23. doi: 10.1016/j.ejogrb.2013.02.001. Epub 2013 Mar 5. PMID: 23465469. |  | x |  |  |  |
| Page L.M | A novel cause for abnormal liver function tests in pregnancy and the puerperium: Non-alcoholic fatty liver disease | Page LM, Girling JC. A novel cause for abnormal liver function tests in pregnancy and the puerperium: non-alcoholic fatty liver disease. BJOG. 2011 Nov;118(12):1532-5. doi: 10.1111/j.1471-0528.2011.03070.x. Epub 2011 Aug 22. PMID: 21880110. |  | x |  |  |  |
| Yogeswaran K. | Chronic hepatitis B in pregnancy: unique challenges and opportunities. | Yogeswaran K, Fung SK. Chronic hepatitis B in pregnancy: unique challenges and opportunities. Korean J Hepatol. 2011 Mar;17(1):1-8. doi: 10.3350/kjhep.2011.17.1.1. PMID: 21494071; PMCID: PMC3304622. |  | x |  |  |  |
| Conover C.A. | Longevity and age-related pathology of mice deficient in pregnancy-associated plasma protein-A | Conover CA, Bale LK, Mader JR, Mason MA, Keenan KP, Marler RJ. Longevity and age-related pathology of mice deficient in pregnancy-associated plasma protein-A. J Gerontol A Biol Sci Med Sci. 2010 Jun;65(6):590-9. doi: 10.1093/gerona/glq032. Epub 2010 Mar 29. PMID: 20351075; PMCID: PMC2869530. |  | x |  |  |  |
| Arfaoui D. | Hepatitis B and pregnancy | Arfaoui D, Fkih M, Hafsa AE, Kaabia N, Azzouz M. Hépatite virale B et grossesse [Hepatitis B and pregnancy]. Tunis Med. 2010 Jun;88(6):383-9. French. PMID: 20517846. |  | x |  |  |  |
| Chen C.-Y | Hepatitis B and pregnancy, the scientific basis for perinatal prevention | Chen C.-Y, Chang M.-H. Hepatitis B and pregnancy, the scientific basis for perinatal prevention  Fetal and Maternal Medicine Review, 2010 21(2), 89-113. doi:10.1017/S0965539510000021 |  | x |  |  |  |
| Adriaens V | HIV in pregnancy: A retrospective study | Adriaens, V., Pexsters, A., Devlieger, R., Vandermeersch, E., & Van De Velde, M. (2009). HIV in pregnancy; aa retrospective study. *International Journal of Obstetric Anesthesia*, *18*. |  | x |  |  |  |
| Kumar A. | HCV infection and pregnancy | Kumar A. HCV infection and pregnancy Salud(i)Ciencia 2007 15:4 (729-731) |  | x |  |  |  |
| Giles M., | Hepatitis C and pregnancy: An update | Giles M, Hellard M, Sasadeusz J. Hepatitis C and pregnancy: an update. Aust N Z J Obstet Gynaecol. 2003 Aug;43(4):290-3. doi: 10.1046/j.0004-8666.2003.00083.x. PMID: 14714713. |  | x |  |  |  |
| Burns D.N | Hepatitis C: Screening in pregnancy | Burns DN, Minkoff H. Hepatitis C: screening in pregnancy. Obstet Gynecol. 1999 Dec;94(6):1044-8. doi: 10.1016/s0029-7844(99)00488-3. PMID: 10576199. |  | x |  |  |  |
| Magriples U | Hepatitis in pregnancy | Magriples U. Hepatitis in pregnancy. Semin Perinatol. 1998 Apr;22(2):112-7. doi: 10.1016/s0146-0005(98)80043-8. PMID: 9638905. |  | x |  |  |  |
| Figueroa Damián R. | Hepatitis B in pregnancy: clinical and prophylactic implications | Figueroa Damián R, Sánchez Fernández L, Benavides Covarrubias E. Hepatitis viral tipo B en el embarazo: implicaciones clínicas y profilácticas [Hepatitis B in pregnancy: clinical and prophylactic implications]. Ginecol Obstet Mex. 1995 Feb;63:90-5. Spanish. PMID: 7698681. |  | x |  |  |  |
| Tompkins R.K | Angiomyolipoma of the liver presenting in pregnancy | Imagawa DK, Lien JM, Dugan MC, Tompkins RK. Angiomyolipoma of the liver presenting in pregnancy. Am Surg. 1994 Nov;60(11):824-6. PMID: 7978673. |  | x |  |  |  |
| Levy | Hepatitis B vaccine in pregnancy: Maternal and fetal safety | Levy M, Koren G. Hepatitis B vaccine in pregnancy: maternal and fetal safety. Am J Perinatol. 1991 May;8(3):227-32. doi: 10.1055/s-2007-999384. PMID: 1827584. |  | x |  |  |  |
| Devriendt K., | Primary structure of pregnancy zone protein. Molecular cloning of a full-length PZP cDNA clone by the polymerase chain reaction | Devriendt K, Van den Berghe H, Cassiman JJ, Marynen P. Primary structure of pregnancy zone protein. Molecular cloning of a full-length PZP cDNA clone by the polymerase chain reaction. Biochim Biophys Acta. 1991 Jan 17;1088(1):95-103. doi: 10.1016/0167-4781(91)90157-h. PMID: 1989698. |  | x |  |  |  |
| Mancal P | The importance of screening for hepatitis B surface antigens in pregnancy | Mancal P, Podrouzek P, Kozlová J, Labohá J. Význam skríninku povrchového antigenu viru hepatitidy B u tĕhotných [The importance of screening for hepatitis B surface antigens in pregnancy]. Cas Lek Cesk. 1989 Nov 24;128(48):1517-20. Czech. PMID: 2611846. |  | x |  |  |  |
| Podrouzek P. | Routine screening for serological markers of viral hepatitis B in pregnancy | Podrouzek P, Mancal P, Kozlová J, Labohá J. Rutinní screening sérologických markerů viru hepatitidy B u tĕhotných [Routine screening for serological markers of viral hepatitis B in pregnancy]. Cesk Gynekol. 1989 Sep;54(8):575-80. Czech. PMID: 2582501. |  | x |  |  |  |
| Arevalo J.A. | Hepatitis B in pregnancy | Arevalo JA. Hepatitis B in pregnancy. West J Med. 1989 Jun;150(6):668-74. PMID: 2665319; PMCID: PMC1026704. |  | x |  |  |  |
| Buffet C | Viral hepatitis during pregnancy and maternal foetal transmission of HB virus | Buffet C. Viral hepatitis in pregnancy and materno-fetal transmission of the B virus. Presse Medicale 1985 Feb;14(7):419-422. PMID: 3157131. |  | x |  |  |  |
| Rana | Hepatitis C Virus Infection in Pregnancy and Children: Its Implications and Treatment Considerations with Directly Acting Antivirals: A Review | Rana R, Dangal R, Singh Y, Gurung RB, Rai B, Sharma AK. Hepatitis C Virus Infection in Pregnancy and Children: Its Implications and Treatment Considerations with Directly Acting Antivirals: A Review. JNMA J Nepal Med Assoc. 2021 Sep 11;59(241):942-953. doi: 10.31729/jnma.5501. PMID: 35199739; PMCID: PMC9107891. |  | x |  |  |  |
| Kang Kook Choi | Hepatocellular carcinoma during pregnancy: is hepatocellular carcinoma more aggressive in pregnant patients? | Choi KK, Hong YJ, Choi SB, Park YN, Choi JS, Lee WJ, Kim KS. Hepatocellular carcinoma during pregnancy: is hepatocellular carcinoma more aggressive in pregnant patients? J Hepatobiliary Pancreat Sci. 2011 May;18(3):422-31. doi: 10.1007/s00534-010-0345-6. PMID: 21116657. | x |  |  |  |  |
| Chu k. | Management of pregnancy-associated hepatocellular carcinoma: A case series | Chu, K. K. W., Chan, A. C. Y., Cheung, T. T., Chok, K. S. H., Lo, C. M., & Poon, R. T. P. Management of pregnancy-associated hepatocellular carcinoma: a case series. 2016 Liver Cancer. | x |  |  |  |  |
| Cao M.K., | [Primary hepatocellular carcinoma with pregnancy and delivery: a case report]. | Cao, M. K., & Han, G. R. Zhonghua gan zang bing za zhi = Zhonghua ganzangbing zazhi = Chinese journal of hepatology, 2010 18(8), 630. https://doi.org/10.3760/cma.j.issn.1007-3418.2010.08.021 | x |  |  |  |  |
| Yoshida Y., | A resected case of hepatocellular carcinoma during pregnancy | Yoshida Y., Yamanaka J., Iimuro Y., Hirano T., Saito S., Nishigami T., Tujimura T., Iijima H., Nishiguchi S., Fujimoto J. A resected case of hepatocellular carcinoma during pregnancy Acta Hepatologica | x |  |  |  |  |
| Haring | Behavior and complications of hepatocellular adenoma during pregnancy and puerperium: a retrospective study and systematic review. | Haring MPD, Spijkerboer CS, Cuperus FJC, et al. Behavior and complications of hepatocellular adenoma during pregnancy and puerperium: a retrospective study and systematic review. *HPB (Oxford)*. 2021;23(8):1152-1163. doi:10.1016/j.hpb.2021.04.019 |  | x |  |  |  |
| Pakkala | Primary Hepatic Choriocarcinoma with Pregnancy: A Diagnostic and Therapeutic Challenge | Pakkala AK, Nekarakanti PK, Nagari B, Bansal AK, Shroff G, Uppin MS. Primary Hepatic Choriocarcinoma with Pregnancy: A Diagnostic and Therapeutic Challenge. *Korean J Gastroenterol*. 2023;81(2):91-94. doi:10.4166/kjg.2022.116 |  | x |  |  |  |
